# Non-genetic inheritance via the male germline in mammals

**DOI:** 10.1098/rstb.2018.0118

**Published:** 2019-02-25

**Authors:** Faye A. Baxter, Amanda J. Drake

**Affiliations:** 1 Royal Hospital for Sick Children, 9 Sciennes Road, Edinburgh EH9 1LF, UK; 2 University/British Heart Foundation Centre for Cardiovascular Science, The Queen's Medical Research Institute, University of Edinburgh, 47 Little France Crescent, Edinburgh EH16 4TJ, UK

**Keywords:** transgenerational epigenetic inheritance, intergenerational epigenetic inheritance, DNA methylation, histones, sRNA, developmental origins of health and disease

## Abstract

Numerous studies in humans and in animal models have demonstrated that exposure to adverse environmental conditions in early life results in long-term structural and functional changes in an organism, increasing the risk of cardiometabolic, neurobehavioural and reproductive disorders in later life. Such effects are not limited to the first generation offspring but may be transmitted to a second or a number of subsequent generations, through non-genomic mechanisms. While the transmission of ‘programmed’ effects through the maternal line could occur as a consequence of multiple influences, for example, altered maternal physiology, the inheritance of effects through the male line is more difficult to explain and there is much interest in a potential role for transgenerational epigenetic inheritance. In this review, we will discuss the mechanisms by which induced effects may be transmitted through the paternal lineage, with a particular focus on the role of epigenetic inheritance.

This article is part of the theme issue ‘Developing differences: early-life effects and evolutionary medicine’.

## 1. Introduction

Numerous studies in humans and in animal models have demonstrated that although development is highly regulated, embryos remain sensitive to environmental cues, and exposure to adverse environmental conditions may result in long-term structural and functional changes, increasing the risk of cardiometabolic, neurobehavioural and reproductive disorders in later life [1]. These ideas have led to the rapid growth of the Developmental Origins of Health and Disease (DOHaD) field. More recently, studies have shown that these effects are not limited to the first generation (F1) but may be transmitted to a second (F2) or a number of subsequent generations, through non-genomic mechanisms [2–4]. For example, human epidemiological studies have demonstrated evidence for such effects on birth weight and cardiovascular risk [5–8]. Such human studies are obviously difficult because of the time scales involved and the influence of genetic, social and cultural factors, and animal studies have been undertaken to better understand the underlying mechanisms [9–11]. These confirm that diverse prenatal insults appear to influence the health of future generations, with effects on cardiometabolic risk factors, reproductive health and neurodevelopment/behaviour [12]. Understanding how effects in one individual may affect multiple subsequent generations is clearly important, and there are implications for health promotion strategies, such that improving health in one generation may significantly improve the health prospects of subsequent generations.

In terms of mechanisms, the transmission of induced, or ‘programmed’, effects on the phenotype through the maternal line could occur as a consequence of multiple influences, for example, through re-exposure via programmed alterations in maternal physiology, such as higher maternal blood pressure, increased maternal glucocorticoid levels, maternal glucose/insulin dyshomeostasis or altered maternal size [13]. Alternatively, programmed changes in maternal care behaviour may lead to the reproduction of the same/similar phenotypes in her children [14]. Although the inheritance of effects through the male line has been described in many studies (for example, see [9,15–19]), this is more difficult to explain, particularly because the influence of the male tends to be limited to the periconceptual period in many animal models. As such, it has been suggested that paternal transmission of a phenotype occurs through effects in the germline (i.e. transmissible through sperm), because in general, in these models, the male contributes little else to the offspring and its environment. In this review, we will discuss potential mechanisms by which induced effects may be transmitted through the paternal lineage, with a particular focus on the role of epigenetic inheritance.

## 2. Intergenerational and transgenerational effects

While the terms ‘intergenerational’ and ‘transgenerational’ have frequently been used interchangeably in the literature, here we will use the following generally accepted definitions. First, ‘intergenerational effects' refers to the inheritance of characteristics between two generations where the developing germline has also been exposed to the same environmental insult. Examples include when a pregnant female (F0) is exposed to an insult, this may result in direct effects on her developing F1 offspring and in addition, the developing germline, which will become the F2 generation, is also exposed. Similarly, when an adult male (F0) is exposed to an insult, the germ cells that will form the F1 generation are also exposed directly. The term ‘transgenerational effects’ is used to describe the transmission of effects across multiple generations where the germline has not been directly exposed. Thus, in the examples given above, transmission to the F3 generation through the female line and to the F2 through the male line.

## 3. Epigenetic inheritance and the barrier of epigenetic reprogramming

Perhaps surprisingly, there is no universal definition of the term ‘epigenetic’, and as such, the term means different things to different people—the changing definitions and usage of the term have been recently discussed in a useful review by Lappalainen & Greally [20]. In studies in the DOHaD field, the term has often been used very broadly to include DNA methylation, histone modifications and/or non-coding RNA. Over the past decade, many animal and human studies reporting associations between the early life environment and later phenotypes have reported differences in DNA methylation, histone modification and/or non-coding RNA (reviewed in [21]), such that it has almost become accepted dogma that the early life environment influences health through induced changes in these marks, despite a general lack of mechanistic evidence.

Leading on from this, a growing number of studies have suggested that ‘epigenetic inheritance’ may be the mechanism by which environmentally induced phenotypic changes can influence progeny [2,22,23]. Epigenetic inheritance is known to occur in plants, in which the germline arises from somatic cells late in the life cycle [22]. The mechanisms include the germline inheritance of DNA methylation patterns (reviewed in [24]) and a role for small RNAs (sRNA) that can target the epigenetic machinery to initiate and maintain transcriptional silencing that can be transmitted to subsequent generations in the absence of the initiating RNA [22]. There is also substantial evidence for epigenetic inheritance in the nematode *Caenorhabditis elegans*, in which the germline is specified at the zygote stage [25,26]. Again, this involves RNAi-based mechanisms, resulting in gene silencing in both soma and germline and these effects can be transmitted, so that the organism maintains a memory of induced changes in gene expression patterns for many generations [27]. For example, piwi-interacting RNAs (piRNAs) can initiate highly stable, heritable epigenetic silencing in the germline that can persist for at least 20 generations [26]. Once established, this long-term memory becomes independent of the piRNA trigger but remains dependent on the nuclear RNAi/chromatin pathway [26] and may mediate the transmission of environmentally induced effects across generations [28,29]. Also in *C. elegans*, double-stranded RNA can be transferred from neurons to the germline and cause transgenerational gene silencing [30].

In mammals, a major barrier to the transmission of ‘epigenetic marks’ across generations is the phenomenon of epigenetic reprogramming that occurs in the germline to ensure the totipotency of the zygote. While reprogramming of DNA methylation occurs in the plant germline and embryo, this is incomplete, facilitating transgenerational inheritance [31]. By contrast, in mammals, extensive reprogramming of the epigenome is thought to be essential to remove potential epimutations and to erase parental imprints. Epigenetic reprogramming occurs during two distinct developmental phases [32]: first, DNA methylation in primordial germ cells (PGCs) is erased during early development in both males and females following the migration of PGCs into the genital ridge [33]. Although this process occurs over the majority of the genome, such that the overall DNA methylation level is reduced by more than 90%, DNA methylation is maintained at some potentially deleterious retroelements, including intracisternal A particle (IAP) retrotransposons, at some other repetitive elements and at a number of single copy genes, including regions of the genome that have been associated with metabolic and neurological disease [34–36]. This is followed by the re-establishment of DNA methylation marks, which occurs during late gestation in males (at least in rodents) and postnatally in females [33,37]. This process is accompanied by extensive remodelling of histone modifications [33]. A further wave of genome-wide epigenetic reprogramming occurs in the zygote following fertilization, including DNA demethylation and remethylation and chromatin remodelling [38], although imprinted loci (and potentially some other regions) are protected from reprogramming during this phase (reviewed in [39]). These processes of epigenetic reprogramming, which would also ensure the removal of epigenetic marks acquired during development and/or induced by environmental factors, represent a major barrier to epigenetic inheritance. Nevertheless, it is possible that abnormal epigenetic reprogramming occurring as a consequence of environmental influences could persist, resulting in effects in subsequent generations that could be deleterious or beneficial, although this remains an area of considerable controversy [22,40–43]. When considering intergenerational effects as defined above, the developing germline will already be present at the time when the insult is experienced and could therefore also be influenced directly—including disruption of the normal process of epigenetic reprogramming. However, for transgenerational effects, the germline should have undergone a normal round of epigenetic reprogramming in the absence of any environmental insult, suggesting that any induced effects must be preserved, maintained and transmitted to a subsequent generation in the absence of an ongoing environmental influence.

## 4. A role for DNA methylation in epigenetic inheritance?

Cytosine methylation (5-methylcytosine, 5mC) at CpG dinucleotides occurs through the actions of the DNA methyltransferases (Dnmts). 5mC is important in the regulation of gene expression, particularly in the maintenance of transcriptional silencing, and it is particularly found at heterochromatic regions of the genome and over repetitive elements. 5mC is important in the silencing of retrotransposons and endogenous retroviral sequences, in the phenomenon of genomic imprinting and in the inactivation of the X-chromosome. DNA methylation can also occur in non-CpG contexts, including CpA, which may account for a significant proportion of cytosine methylation in some cell types [44]. DNA demethylation can either occur passively through DNA replication or actively through the action of the Ten–eleven translocation methylcytosine dioxygenases (Tets) 1–3 [45,46]. So, what is the evidence that induced abnormalities in DNA methylation can be transmitted to offspring through the male germline?

The term ‘epiallele’ defines an allele that can exist in variable epigenetic states and ‘metastable epialleles' are mammalian alleles at which variable expression associates with epigenetic differences. Thus, the stochastic establishment of DNA methylation at metastable epialleles during early development can lead to differences in epigenetic signatures between individuals. Metastable epialleles have been described in mice, notably the murine *agouti viable yellow* (*A^vy^*) gene. The wild-type *agouti* gene encodes a molecule producing either black eumelanins (*a*) or yellow phaeomelanin (*A*). Transient *A* expression during a specific stage of hair growth results in a sub-apical yellow band, resulting in the brown (agouti) coat colour of wild-type mice. The insertion of an IAP into the agouti gene produced the *A^vy^* metastable epiallele, and the resulting ectopic gene expression results in obesity, yellow fur colour and the development of tumours. Methylation of the 5′ long-terminal repeat (LTR) of the *A^vy^* IAP correlates with gene transcription and the stochastic establishment of DNA methylation at the LTR during development leads to a variable phenotype even among isogenic littermates. Importantly, studies showed that the availability of methyl donors in the maternal diet during gestation can impact on the phenotype of the offspring [23], supporting the concept that DNA methylation at metastable epialleles is labile and can be influenced by the environment at a critical stage of development. In the *Axin^Fu^* mouse, an IAP element incorporated within *Axin^Fu^* leads to the transcription of a form of Axin that associates with the development of a kinked tail. IAP methylation correlates with tail kinkiness and maternal methyl donor supplementation can affect the tail phenotype of the offspring [47]. In the *A^vy^* strain, the phenotype is transmissible through the maternal line to her offspring [48]; however, studies suggest that DNA methylation at the *A^vy^* locus is erased and re-established normally between generations, so that rather than differences at this locus being transmitted directly, other mechanisms must be responsible for the similarity of DNA methylation patterns between parents and offspring [49]. Epigenetic inheritance has also been shown with *Axin^Fu^* but this is influenced by strain background [50]. Whether these phenomena exist in other mammals, particularly in humans, is unclear; indeed, several potential epialleles causing human disease have been found to be dependent on DNA sequence polymorphisms, so that the aberrant gene silencing (epimutation) is established anew in each generation after normal germline epigenetic reprogramming [51].

*In utero* exposure to undernutrition, including unbalanced maternal nutrition, is commonly used to induce programmed effects including alterations in birth weight, adiposity, glucose/insulin homeostasis and behaviour. For example, in a mouse model of maternal undernutrition, the first generation (F1) offspring of undernourished dams have low birth weight, altered adiposity and later glucose intolerance and these effects were transmissible through the paternal line to a second (F2) generation [3]. In the F1 male offspring, extensive profiling of germline DNA methylation identified hypomethylation at a number of differentially methylated regions (DMRs) enriched at nucleosome-containing regions [3]. Although this differential methylation was not maintained in F2 offspring brain or liver, the expression of a number of neighbouring genes was altered, suggesting that although DNA methylation does not directly affect gene expression at these loci, there may be long-term effects of altered DNA methylation in early development [3]. Other environmental insults that have been shown to induce programmed effects include ‘endocrine disruptors’—chemicals that can interfere with endocrine systems—and a high-profile series of studies using the fungicide vinclozolin has reported inter- and transgenerational effects on a number of health parameters in rats, in association with alterations in DNA methylation in the sperm of multiple generations (for example [11,19,52]). Whether this phenomenon is widespread is unclear, indeed a recent detailed study in mice showed negligible effects of vinclozolin exposure on *de novo* DNA methylation and only subtle transcriptional changes in F1 prospermatogonia, which were not seen in a second generation [53]. Furthermore, the fact that the transgenerational effects of vinclozolin differ between inbred and outbred strains of rats suggests that genetic, rather than epigenetic variation could be responsible [54]. In a well-characterized rat model, we have shown that *in utero* glucocorticoid overexposure associates with low birth weight and glucose intolerance in the exposed F1 offspring in association with intergenerational effects—the phenotype is transmissible to the F2 generation through both maternal and paternal lines [9,10,16]. We found altered gene expression and DNA methylation at candidate imprinted genes in liver from F1 and F2 offspring of glucocorticoid-treated females; however, notably, the direction of the changes in gene expression and the location of DNA methylation changes differed between the two generations [16]. Furthermore, using both methylated DNA immunoprecipitation-sequencing and enhanced reduced representation of bisulfite sequencing to profile DNA methylation in the germline and sperm of F1 males, we were unable to detect any differences between glucocorticoid-exposed and control males [10]. Furthermore, although in the *A^vy^* model, feeding pregnant dams a diet rich in methyl donors during pregnancy was associated with a shift in DNA methylation at the *A^vy^* locus in her F1 offspring [23], maternal diet-induced *A^vy^* hypermethylation was not transmitted across generations, despite the established precedent for intergenerational effects in this mouse strain [55].

Early postnatal exposures can also lead to effects on behaviour and metabolic health, with inter- and transgenerational consequences. Exposure to chronic unpredictable stress during the early postnatal period results in altered behaviour in exposed male mice in adulthood and in their offspring [56]. In this model, there were very small changes in DNA methylation upstream of the transcription initiation site of candidate genes in the germline of exposed males [56,57]. Notably, some of these differences in DNA methylation occurred within CpG islands, areas of the genome that are generally maintained in a hypomethylated state. There were also small changes in CpG methylation in the same genic regions in the offspring brain, which were present at some, but not all of the same CpGs, and at some additional sites [56,57]. Also in mice, the offspring of males maintained on a low protein diet following weaning had altered hepatic expression of a number of genes important in lipid metabolism and modest changes in hepatic DNA methylation when compared to offspring of males on control diet, although there were no differences in DNA methylation in sperm [58]. In another mouse model, exposure of adult (F0) males to an odorant stressor is associated with effects on the behaviour of two subsequent generations (F1 and F2), and detailed experiments using *in vitro* fertilization and cross-fostering suggest that the transmission of these effects occurs through the gametes [59]. Specifically, small changes in DNA methylation at a single CpG were identified at the 3′ end of a candidate locus (an odorant receptor) in the sperm of F0 exposed males and at the same and one additional CpG in their F1 offspring, although this was not seen in the crucial areas of the brain thought to be responsible for the behavioural phenotype [59]. Finally, in mice, exposure of males to chronic unpredictable stress in early life results in effects on behaviour in adulthood, with effects on some, but not all of the same behaviours in males but not females in the next (F1) generation and effects in females but not males in the F2 generation [60]. However, very small differences in DNA methylation were reported at a single gene in F1 male sperm—this occurs at a region where DNA methylation appears to be in the range 1–3%—such that the biological relevance of these differences, and how they might result in sex-specific effects in the offspring, are unclear.

Although these and other studies suggest that early life exposure to insults can lead to effects on DNA methylation in sperm in association with the transmission of a phenotype to the next generation, the extent to which these changes in DNA methylation are responsible for this transmission is unclear. In these models, the penetrance of the phenotype is high—indeed, effects are found using very small numbers of animals—but the percentage DNA methylation changes that are reported in sperm are very low [56,59]. DNA methylation at any individual CpG in haploid sperm is binary (i.e. a single sperm will carry either a methylated or unmethylated CpG at that locus), so that small changes in DNA methylation in a population of sperm reflect altered DNA methylation in a small proportion of sperm only. This does not fit well with specific outcomes that depend on fertilization by a single sperm. Additionally, the observed effects on gene expression in the offspring tissue(s) of interest often occur in the absence of detectable changes in DNA methylation [3,61]. This suggests that direct transmission of changes in DNA methylation is unlikely to be the underlying mechanism for the transmission of the phenotype, at least in these models [3,61]. Furthermore, recent studies showing that the effects of interindividual ‘epivariation’ exert a stronger influence on the sperm epigenome than environmental exposures—for example, stochastic epigenetic variation affects the mouse sperm methylome to a greater extent than diet—suggest that factors other than DNA methylation may account for the transmission of environmental effects on the phenotype to the offspring [62,63].

## 5. Histone modifications

During the final stages of mammalian spermatogenesis, most histones are replaced by sperm-specific protamines; however, a small percentage of histones are retained at key loci in mature sperm [64] and some studies have suggested that alterations in sperm histones may underpin the transgenerational transmission of phenotypes [65,66]. Disruption of histone methylation by overexpressing the KDM1A histone lysine 4 demethylase results in the loss of the histone mark H3K4me2 and changes in sperm RNA content, in association with an increased rate of birth defects, neonatal mortality and altered gene expression in the offspring [65]. There are some reports of alterations in sperm histones in animal models as a consequence of environmental exposures that associate with intergenerational effects, including dietary challenge and drug administration. In mice, consumption of a high-fat diet is associated with altered histone H3 occupancy at key genes and changes in H3K4me1 enrichment at transcription regulatory genes in sperm and with altered expression of some candidate genes in offspring liver [66]. Cocaine administration in rats results in changes in histone modifications specifically at the brain-derived neurotrophic factor (*Bdnf*) locus in sperm [67]. Although this was associated with altered *Bdnf* gene expression in the medial prefrontal cortex, this effect was only seen in male offspring. Additionally, histone acetylation was altered at the *Bdnf* locus in the male brain, but whether this was also the case in the female brain was not reported [67]. Induction of hepatic damage using the hepatotoxin carbon tetrachloride results in intergenerational effects on liver fibrosis and this occurs in association with alterations in histone methylation at a candidate gene—the antifibrogenic factor peroxisome proliferator activated receptor *γ* (PPAR*γ*) [68].

It is not known how changes in histone modifications in sperm avoid the considerable remodelling of modified histones that occurs in the early embryo and persists at specific gene loci and in specific cells/tissues in the adult offspring (and often in a sex-specific manner). Thus, further studies are required to delineate the mechanism(s) by which induced alterations in histones lead to the transmission of phenotypes and the importance of this phenomenon in mammals. Indeed, in contrast to studies focusing on candidate loci, in our studies in which we undertook detailed genome-wide profiling of activating, repressive and enhancer-associated histone modifications in the glucocorticoid-programmed rat model, we identified no differences between sperm from glucocorticoid-exposed and control males [10].

## 6. Small RNAs

Studies in plants and in *C. elegans* suggest that sRNAs are important in the triggering of heritable gene silencing [22,28,29]. In mammals [18,62,69], mature sperm carries a significant population of sRNAs including miRNA, piRNA, tRNA-derived small RNAs (tRNAs) and repeat associated sRNAs, all of which may be important in the post-fertilization zygote [70,71]. Accordingly, a growing number of studies have suggested that alterations in sRNA might be important in the inter- and/or transgenerational transmission of induced effects through the male germline. In rodent studies, paternal consumption of a high-fat diet has been linked to altered expression of sperm miRNAs in some [69,72], but not all studies [73]. Exposure of pregnant female mice to vinclozolin leads to the specific dysregulation of miRNA in PGCs, with downstream effects on PGC differentiation, an effect that persists for three generations [74]. Early postnatal stress exposure has been reported to lead to altered expression of sperm miRNAs [75,76], although we were unable to find any changes in sRNAs in the germline following *in utero* glucocorticoid overexposure in rats, despite performing deep sequencing and candidate gene analysis of miRNAs that were altered in other models. Recent studies have suggested a role for tRNAs. Protein restriction in mice has been found to affect sRNA levels in mature sperm, with increased levels of tRNA fragments, which are delivered into sperm by epididymosomes during maturation [62]. In mice, paternal exposure to a high-fat diet up to six months of age associates with metabolic dysfunction in the offspring and this effect is reported to be mediated by sperm tRNAs [18]. Although the authors suggest that sperm tRNA might lead to metabolic dysfunction in adulthood by affecting metabolic gene expression through a transcriptional cascade effect, the precise mechanisms by which this is targeted specifically to pancreatic islet cells remain unclear. Recent data from this, and other groups, suggest a key role for the tRNA methyltransferase Dnmt2 in this process [77,78]: deletion of Dnmt2 abolished the sRNA-mediated transmission of the high-fat diet-mediated metabolic dysfunction in the offspring. Furthermore, Dnmt2-mediated post-transcriptional RNA modifications may impact on the biological properties of sRNAs and it is possible that these modifications are of particular importance in the transmission of effects through the germline [18].

## 7. Potential alternative mechanisms

Although the potential importance of ‘epigenetic inheritance’ is of great interest, questions remain about its relative importance in mediating the transmission of programmed effects across generations in mammals. So, what other explanations might there be? DNA damage in sperm may affect offspring development [79] and exposure to environmental insults may affect sperm motility, morphology and function [80]. Alternative mechanisms include the actions of factors in seminal fluid [81], sperm exosomes [82], microbiome transfer (to the mother during mating) or the transmission of metabolites [22,83]. Studies suggest that the prior experience of the father can influence the behaviour of the mother [84,85] so that paternally induced maternal effects in the offspring could be important in mediating trans- and intergenerational effects (reviewed in [86]). For example, differences in mate quality may affect a mother's investment in her offspring's growth and development, either to maximize the survival of ‘high-quality’ offspring or to improve the survival of offspring from ‘lower-quality’ fathers. Indeed, in mice, females mated with males that have experienced social enrichment invest more time nursing their offspring [87]. The current interest and focus on epigenetic inheritance have meant that many of these alternative mechanisms for the transmission of phenotypes across generations have been somewhat neglected.

## 8. Conclusion

A large number of studies have shown that the early life environment influences health and disease risk and a growing number of reports suggest that these effects occur in association with alterations in DNA methylation, histone modifications and/or sRNAs. Such effects are also associated with alterations in the germline epigenome and have resulted in the suggestion that the transmission of effects across generations occurs as a result of transgenerational epigenetic inheritance. Despite the substantial interest that has been generated in this area, the data in support of transgenerational epigenetic inheritance in mammals remain limited. Further studies are necessary to understand whether (and how) induced alterations in the germline epigenome can escape the barrier of epigenetic reprogramming in the germline and following fertilization and to delineate the mechanisms by which small alterations in the sperm epigenome might lead to complex, tissue/cell-type specific and often sex-specific effects in offspring.

## Data accessibility

This article has no additional data.
